# Patient- and person-reports on healthcare: preferences, outcomes, experiences, and satisfaction – an essay

**DOI:** 10.1186/s13561-016-0094-6

**Published:** 2016-05-21

**Authors:** K. Klose, S. Kreimeier, U. Tangermann, I. Aumann, K. Damm, on behalf of the RHO Group

**Affiliations:** 1 Health Economics and Health Care Management, University of Bielefeld, P.O. Box 10 01 31, 33501 Bielefeld, Germany; 2 Center for Health Economics Research Hannover (CHERH), Leibniz University Hannover, Otto-Brenner-Straße 1, 30159 Hannover, Germany; 3 Center for Health Economics Research Hannover (CHERH), Hannover Medical School, Carl-Neuberg-Straße 1, 30625 Hannover, Germany

**Keywords:** Patient reports, Patient-reported outcomes/PRO, Experiences, Satisfaction, Preference

## Abstract

With the shift towards patient-centered healthcare, patient- and person-reports of health-related factors, including outcomes, are seen as important determinants for evaluating and improving healthcare. However, a comprehensive, systematic categorization of patient- and person-reports is currently lacking in the literature. This study aims at developing a new classification system with well-defined constructs for patients’ and persons’ self-reports on health and healthcare. A literature research and evaluation by the Reported Health Outcomes (RHO) Group were used to develop this classification system. The new classification system includes patient- and person-reported preferences, outcomes, experiences, and satisfaction related to healthcare and health outcomes. Moreover, the most constitutive methods to measure these four categories – preferences, outcomes, experiences, and satisfaction – have been described in this article. Even though the value of patients’ and persons’ perspectives on healthcare is increasingly being recognized, its measurement and implementation presents a lasting challenge to researchers, clinicians, patients, and the general population.

## Background

### Patient- and person-reports: differing outcomes and aspects

Patient-reported factors including patients’ adherence to treatment, satisfaction with treatment, and experienced health outcomes – as determined by patient preferences – are increasingly seen as important for clinical adherence and uptake of healthcare interventions [1]. In this context, the terms patient-reported outcome (PRO) or patient-reported outcome measure (PROM) are frequently used to evaluate healthcare systems. However, PRO is a multifarious term and a comprehensible categorization is lacking, even though several methods are used to measure patient-reports. This study attempts to clarify the term ‘PRO’ and methods used for measuring PROs.

PRO is frequently used as an umbrella term for health outcomes that are directly and subjectively reported by patients [2]. The Food and Drug Administration (FDA) defines PRO as “any report of the status of a patient’s health condition that comes directly from the patient, without interpretation of the patient’s response by a clinician or anyone else” [3]. Hence, PROs measure health status from the patient’s perspective. PRO has also been defined more broadly, addressing information about health conditions and its management [4]. Thus, definitions differ considerably and continue to be used inconsistently.

To clarify PRO content, several classification systems were developed (e.g., by Valderas and Alonso [5]), many of which are based on specific constructs despite an universal agreement that PRO constructs do not exist. Symptoms, functioning, general health perception, well-being, Health-Related Quality of Life (HRQoL), patient satisfaction, preferences, adherence to treatment and other elements of healthcare and its results have been defined in this context [2–4, 6–8]. However, the terms PRO and outcome are ambiguous, rendering it difficult to determine PRO constructs. Within healthcare, the term outcome refers to end results or consequences of treatment, interventions, or healthcare [9]. However, it is debatable whether adherence to treatment can be regarded as a consequence of treatment. Adherence is an aspect of the therapy, process, and management involved in healthcare that helps to achieve the treatment’s results [10]. Moreover, preference might not solely be seen as an outcome; individuals are often asked about their treatment or health status preferences without any prior experience. Furthermore, there is a fierce discussion on whether PROs should be self-reports or whether others’ assessments (e.g., close relatives) may be considered as PROs [6, 8]. Thus, the term PRO is used in different contexts and can be interpreted differently.

## Methods

Based on this lack of clarity, we aimed to develop a new classification system for different aspects of health- and healthcare-related reports obtained directly from a patient or a person. A literature research, evaluated by a group of interdisciplinary researchers (health economics, public health and economics) – the Reported Health Outcomes (RHO) Group – was used to develop this classification system. Additionally, the most constitutive methods to measure the categories of the classification system are described here.

## Results

### A new classification system for patient- and person-reports on health and healthcare

**Fig. 1:**
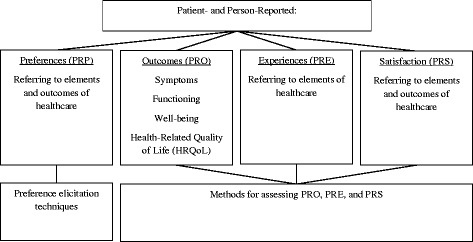
Classification of patient- and person-reports about health and healthcare

A new concept for organizing single terms on health and healthcare-related patient- and person-reports is illustrated in Fig. 1. As many different constructs can be reported by patients or persons themselves, the generic term *patient- or person-reports* was chosen and revised to patient- and person-reports to give equal weightage to both sources. The inclusion of *person* is relevant when considering surveys with the general population on health and health-related preferences that are used to develop preference-based measures of HRQoL [11–13]. Moreover, the term person also acknowledges the value of proxy-reports given by relatives, caregivers, or other health professionals when the patient is unable to comment on his/her health: the proxy’s assessment from the patient’s perspective (proxy-patient perspective) and/or the proxy’s own assessment of the patient (proxy-proxy perspective). The inability of a proxy to fully comprehend the patient’s view and the resulting difference between proxy-report and self-report, the so-called inter-rater gap, is often argued in literature (e.g., lack of validity) [6, 14, 15]. Whenever possible, the patients should report themselves to allow for unbiased results. However, self-assessment can be challenging or even impossible for some patient groups (e.g., frail elderly, cognitively impaired, advanced disease, very young children) [14–16]. In those cases an appropriate proxy has to be identified. So, the proxy assessment can complement or substitute the patient assessment and, thus, makes it possible to include this (possibly missing) information on patient perspectives in health care [14, 15]. Thus, even though the inclusion of proxy-reports as PROs has been previously criticized [3, 8] we regard its inclusion as necessary. Furthermore, the term patient- and person-reports clarifies that the point of view (of the patient or the person) is the only aspect that all categories and constructs have in common.

The various patient- and person-reported constructs can be classified into four categories: *preferences*, *outcomes*, *experiences*, and *satisfaction*, as shown in Fig. 1. *Patient- and person-reported preferences* (PRP) refer to preferences to choose or to prefer one item more than another (e.g., a therapy component). Through PRP, researchers can obtain information regarding the best option or treatment from the respondents’ point of view [1]. The second category, *patient- and person-reported outcomes* (PRO), includes constructs that describe healthcare outcomes. These constructs focus on the patient’s or a person’s health status (e.g., functioning in daily life). The third category, *patient- and person-reported experiences* (PRE), considers constructs regarding patients’ and persons’ experiences assessed during and after healthcare (e.g., about the occurrence of specific events). Hence, in contrast to PRO, PRE describes reports that focus less on a patient’s and person’s health status while emphasize the external care process. *Patient- and person-reported satisfaction* (PRS) includes self-reported satisfaction pertaining to outcomes or elements of healthcare. In contrast to PRE, PRS involves evaluation. However, experiences and satisfaction are often used synonymously in literature [17].

#### Patient- and Person-Reported Preferences (PRP)

*Preferences* are defined as liking something better than another or tendency to choose [18]. The term refers to the relative desirability of something or someone (e.g., healthcare technology), but is conceptualized differently across disciplines. In economics, the desirability of a good or service (e.g., healthcare) can be understood in terms of its utility, a measure of (expected) satisfaction gained from the consumption of a product or service. Thus, preferences are a result of relative subjective assessments of the costs and benefits of alternatives. Preference, utility, and value are often used interchangeable despite their differences [1, 19]. Preference is characterized as an umbrella term. The result of the preference measurement – utility or value – depends on the way of measurement, e.g., the framing of the question. A question framed under certainty will yield values, whereas a question framed under uncertainty will yield utilities [19]. In this article, preferences are broadly defined as values that individuals attach to aspects of health as outcomes and elements of healthcare [1] (see Fig. 1).

Similarly, the term *patient preferences* does not have a clear definition. However, it is generally accepted that patient preferences are statements made by patients regarding the relative desirability of a range of health experiences, treatment options, and health states [20]. In this article, the term patient preferences is used to indicate the value that patients or persons attach to aspects of health as outcomes and elements of healthcare (health-related preferences). Patient preferences refer to the individuals’ evaluation of the dimensions of health outcomes among a large number of preferences that may influence healthcare choices. These judgments are materialized through statements (e.g., during a counselling interview) or actions (e.g., selection of physician or therapy) [1, 20].

Market orientation in the healthcare system leads to higher competition between healthcare organizations that increasingly focus on patients’ needs. Knowledge about preferences of patients and the general population allow healthcare organizations to optimize their consumer- or patient-oriented approach. Interest in measuring and including individual’s preferences in healthcare is growing [1, 21, 22]. This includes the preferences of persons – general population/healthy individuals – as well as patients. Preference elicitation techniques can be used to gauge consumer preferences of health status, healthcare programs, or health technology assessment [23]. Besides the ethical considerations for patient views, there are various reasons for integrating patient preferences in healthcare policy, such as improving treatment uptake and real-world efficiency of healthcare technologies, facilitating consumer empowerment, and advancing shared medical decision-making. For example, measuring patient preferences can help clinicians making decisions that are consistent with patient preferences (patient-centered healthcare), thereby improving health outcomes [1, 21].

#### Patient- and Person-Reported Outcomes (PRO)

*Outcome* is defined as something that results or follows from an activity or process [18]. In the context of healthcare, outcome is defined as the results or consequences of treatment or healthcare [9]. As depicted in Fig. 1, outcomes comprise the most commonly addressed constructs in PRO literature: symptoms, functioning, well-being, and HRQoL [2, 3, 6, 8, 17]. These four constructs provide information about the patient’s and person’s current health status that can be assessed through self-reports.

*Symptoms* are defined as “subjective evidence of disease or physical disturbance observed by a patient” [18]. In contrast to a sign of a disease (e.g., high cholesterol level), symptoms can only be reported by patients and persons [24].

The construct *functioning* is synonymous with functional status [2, 8]. Functioning includes body functions and body structure, as well as activities and participation. According to the International Classification of Functioning, Disability and Health (ICF), functioning is defined as a “dynamic interaction between a person’s health condition and the contextual factors: environmental and personal factors” [25].

W*ell-being* has been defined inconsistently in existing literature. Its previously accepted definition as “a condition characterized by happiness, health, or prosperity” [18] has been criticized for focusing descriptions or dimensions of well-being instead of its definition [26]. Happiness, life satisfaction, ability to fulfil goals, and positive emotions are the most commonly cited aspects of well-being in the context of health [27–29]. A newer approach defines well-being as “the balance point between an individual’s resource pool and the challenges faced” [8, 26]. Thus, measuring well-being provides information about how a patient or person feels [8].

Similarly, there is no consistent definition of the construct *HRQoL*. It can be defined as a multidimensional construct that includes physical, emotional, mental, social, and behavioral components of well-being and functioning from the point of view of the patient and/or observer [3]. This definition is based on the WHO definition of health [30]. It is important to differentiate HRQoL from the broader construct Quality of Life (QoL) that comprises different components of life (including health) [31].

Collectively, the different PRO constructs are indicators of disease activity and progression and therefore have applications in various fields within the healthcare system [6]. In health economics, PROs are often used to include the patient’s perspective in the evaluation of healthcare and for quality assurance in the healthcare system [2]. In clinical trials, a PRO instrument may be used to measure the patient-relevant outcomes of a medical intervention [3].

#### Patient- and Person-Reported Experiences (PRE)

The word *experience* stems from the Latin experientia, meaning a trial or experiment [18]. This close connection to the word experiment demonstrates the need for real-life observations of the given facts in order to gain new experiences and report about experience. Throughout life, one gains experiences. Thus, a person’s experiences are constantly evolving.

*Patient experience* is a concept frequently applied in the healthcare setting as it provides a report of the healthcare from the receiver’s perspective. Patient-reported experience measures (PREMs) for specific services, events, or the entire treatment process is a detailed report of the patient’s or person’s perspective, offering evidence on areas of improvement or on humaneness of care (i.e. whether the patient is treated with dignity and respect) [17, 32]. Experiences are objective facts of events that occur in relation to an individual; however, the individual’s or observer’s assessment of experience adds valence, which is either positive or negative [33]. An individual’s evaluation of an experience is based on reference values and previous experiences that lead to satisfaction or dissatisfaction.

PREMs are used to explore patients’ and persons’ experiences throughout the care process, rather than at the end of their treatment [34]. Hence, unlike PROs, they are not used to evaluate the outcome of care. These measures include, among others, information given to patients, the extent to which the patient’s social environment was involved in the treatment process, and coordination of care, including transition between sectors and waiting times [17, 35, 36]. Information is identified about different elements of healthcare as presented in Fig. 1. However, patients and persons report on their experience rather than their satisfaction with these elements of healthcare.

#### Patient- and Person-Reported Satisfaction (PRS)

*Satisfaction* is the fulfilment of one’s wishes, expectations, needs, or desires [37]. According to this definition, something that is not desired or anticipated will not lead to satisfaction or dissatisfaction. Satisfaction involves a comparative process in which experiences gained in a specific situation are compared to previously formed expectations [38]; the lower these expectations are, the higher the level of satisfaction, and vice versa.

*Patient satisfaction* has not been clearly defined. Patient satisfaction is a subjective evaluation of medical care by the patient based on the extent to which the patients’ expectations were fulfilled [37]. The main elements that patients can assess subsequently leading to their satisfaction or dissatisfaction are (1) medical treatment, (2) non-medical aspects of treatment (e.g., communication), (3) infrastructure (e.g., technical equipment), and (4) financial factors connected to the treatment [39]. While patient satisfaction is predominantly based on elements of healthcare, it also includes satisfaction with treatment outcomes (see Fig. 1). Satisfaction with healthcare can be measured by asking the patient himself/herself, a person as proxy or by measuring the person’s (as a relative of a patient) own satisfaction with medical care.

Improving the processes and structures of healthcare can lead to increased patient satisfaction [40]. However, there is no consensus on whether patients’ satisfaction reflects the quality of care administered. Inaccuracies are likely because of the cognitive mechanisms outlined above and because patients may perceive elements other than the technical quality of care as important; thus, even poor quality healthcare may be perceived as satisfactory [41]. Hence, patient satisfaction is a more subjective than objective assessment of the quality of care. Nonetheless, patient satisfaction continues to be used extensively to include the patient’s perspective in quality assurance and treatment evaluation [37].

The strong interlinks between satisfaction and experiences and the clear distinction between them are important to note. Satisfaction cannot arise without the experience, but the isolated experience – without being compared with past experiences or expectations – does not equate to satisfaction or dissatisfaction. As satisfaction is based on the comparison of past and present experiences, it always contains some sort of evaluation and is therefore subjective. Reports of experiences are more descriptive, allowing for more objectivity. Due to this important distinction, they are treated as different concepts, illustrated as two separate columns in Fig. 1.

### Measuring patient- and person-reports

After presenting the classification system, we attempt to clarify the methods that can be used to measure patient- and person-reports. Healthcare literature includes a large number of different methods used by researchers. However, the purpose served by each method, their theoretical basis, and practical applications vary. Generally, qualitative, quantitative, or mixed-methods approaches are used in studies measuring PRP, PRO, PRE, and PRS.

In the following section, the methods used for measuring patient- and person-reports are clarified. Because the measurement of preferences is a relatively new approach in healthcare, diverse methods have been used and no complete overview of their distinctive characteristics has been published. Therefore, we first present an overview of preference elicitation techniques (methods measuring PRP). The methods for measuring PRO, PRE, and PRS are then described. For measuring PRO, PRE, and PRS there are no special methods in comparison to PRP methods, but there are also possibilities to differ the methods presented in the following section with examples.

#### Measuring PRP

Various methods to assess patient and person preferences are available. Preference elicitation techniques can be applied within a qualitative approach of data collection through different forms of interviews and quantitative data collection via standardized self-reported questionnaires. Prior to collecting quantitative data on preferences, qualitative pre-studies are often conducted in the first step of a research study with a mixed methods approach (e.g., identifying relevant attributes and levels in focus groups for developing choice sets in discrete choice experiments [DCE]). Besides qualitative and mixed methods approaches, quantitative methods are most frequently used to measure PRP and are therefore the focus of the following section.

Preferences can be elicited using either revealed or stated preference data. Revealed preference data are obtained from real past behavior of consumers (e.g., patients). Stated preference data are collected through surveys, in which respondents consider one or more different hypothetical products or services (e.g., healthcare elements like therapy options) and express their preferences for them [42]. This section of the article focusses on stated preferences. Table 1 presents an overview of stated preference elicitation techniques and describes some characteristics including underlying theories, measurement method, and analysis. Underlying theories were included as it is often considered as a criterion for selecting methods for health surveys. For example, the lack of underpinning economic theory is increasingly being used to critique the application of rating scales for preference measurement. Inclusion of economic theoretical underpinnings ensures the method’s relevance and acceptance in science community, as these theories are the basis for conducting consistent economics evaluations [42, 43]. In Table 1, the theoretical classification of methods was based on a broad understanding of economic theory.

**Table 1:** Stated preference elicitation techniques

Method		Underlying theory	Measurement	Analysis
Contingent valuation	Open-ended [57, 58]	Rooted in welfare economics, namely in the neo-classical concept of economic value based on individual utility maximization. Contingent valuation surveys directly obtain a monetary (Hicksian) measure of welfare associated with providing a good/service.	• Direct query about willingness-to-pay or willingness-to-accept • For example: “Please state the largest amount you are willing to pay for the good/service.”	Various statistical methods depending on study aims (e.g., minimum, maximum, mean, and regression)
	Dichotomous choice [58, 59]		• Dichotomous question with reference to a given price • For example: “Would you be willing to pay *X* € for the good/service?”	Binary choice models (e.g., binary logit, binary probit)
	Bidding game [58]		• Dichotomous question in form of an auction • For example: Would you be willing to pay *X* € for the good/service? Would you be willing to pay *X + Y* € (*X-Y* €) for the good/service?	Various statistical methods depending on study aims (e.g., minimum, maximum, mean, parametric, and non-parametric tests)
Self-explicated approaches [60]		No underlying economic theory	• Unacceptable attributes are removed • The level of each attribute is evaluated on a desirability scale (e.g., 0 the worst level of the attribute and 100 the best) • The respondent is asked to allocate, for example, 100 points across the attributes to reflect their relative importance • In stage 1 or 2, different combinations of comparative or non-comparative methods could be used	• Part-worth: multiplying the importance weights (stage 2) with the attribute and level of desirability ratings (stage 1), additive assumption • Overall utility: sum of the part-worth
Analytic hierarchy process [46, 61]		No underlying economic theory	1. The attributes that contribute to the problem must be identified and arranged in a hierarchy according to aims, attributes, and alternatives 2. Hierarchy levels are assessed by paired comparisons 3. A matrix is created using pairwise ratios and the relative weights are calculated 4. Relative weights of the levels in stage 3 are aggregated	Calculating the relative weights of hierarchy levels with the eigenvector method
Conjoint Analysis	Not choice-based [62]	Depends on the method and approaches used	Variety of methods and approaches, such as rating or ranking of different alternatives	• Interval scaling (e.g., OLS* regressions) • Ordinal scaling (e.g., MONANOVA*, PREFMAP*, LINMAP*, ordered logit-/probit-regressions)
	Choice-based (discrete choice experiment) [43, 63]	Random utility theory	• Choice between two or more discrete alternatives (selection of most preferred alternative) • Alternatives are described by a set of attributes and each attribute takes one of several levels	• Two alternatives in the choice set: binary discrete choice models (e.g., binary logit, binary probit) • Three or more alternatives in the choice set: multiple discrete choice models (e.g., multinomial logit, nested logit, mixed logit, multinomial probit, heteroscedastic extreme value)
Standard gamble [64]		Utility theory by von Neumann and Morgenstern	• Choice between a fixed health status and a lottery with the probability *p* to obtain the best possible outcome and the probability *1 - p* to obtain the worst possible health status • For example: a chronic health state preferred over death: 1. Respondents are offered two alternatives: (A): two possible outcomes; aa) the subject lives in a good health with the probability *p* for a fixed time *t*, or ab) the subject dies immediately with the probability *1 - p* (B): the subject lives in a fixed health status *i* for the rest of his/her life *t* 2. Respondents’ indifference point is located by varying the probability *p*	• For example: chronic health state preferred to death: At indifference point, the required preference score for health state *i* is *h*_*i*_ *= p*
Time trade-off [64, 65]		No underlying economic theory	• Trade-off between life years in a state of less than perfect health and a shorter life span in a state of perfect health • For example: a chronic health state preferred over death: 1. Respondents are offered two alternatives: (A) health state *i* for time *t*, followed by death; (B) full health for time *x < t*, followed by death 2. Respondents’ indifference point is located by varying the time *x*	• For example: chronic health state preferred to death: At indifference point the required preference score for health state *i* is given: *h*_*i*_ *= x/t*
Rating scale [64, 65]		No underlying economic theory	• Direct rating on a line with or without internal markings • For example: a chronic health state preferred to death: 1. Respondents receive information about a batch of chronic health states, age of onset, the age of death, and two reference states (“full health”, “death”) 2. Respondents are usually asked to select the best and the worst of those health states 3. The remaining health states are placed on the rating scale relative to each other	• For example: a chronic health state preferred to death. Preference value for health state is the scale value of its placement

*OLS: ordinary least squares, *MONANOVA*: monotonic analysis of variance, *PREFMAP*: preference mapping, *LINMAP*: linear programming technique for multidimensional analysis of preference

Stated preference elicitation techniques can be classified in different ways. The most widely accepted classification is into Contingent Valuation (CV) and multi-attributive preference methods [42]. CV is a method that directly estimates the respondents’ willingness-to-pay (WTP) or willingness-to-accept (WTA) for goods, services, or negative interferences in healthcare through questionnaires. In a hypothetical market scenario, goods and negative interferences are tradable. The respondents’ WTP/WTA are determined via various question techniques (e.g., open-ended CV, dichotomous choice, or bidding game; see Table 1) [42, 44]. While CV analyzes one attribute of the product at a time, multi-attributive preference methods explore more than one attribute simultaneously. The latter is an umbrella term for modeling preferences for a healthcare product or service that is described in terms of its attributes and levels [42]. There are three different types of multi-attributive preference measurement: (1) direct or compositional preference measurement, (2) de-compositional methods, and (3) a combination of both. For compositional preference measurement, single characters – each attribute and level – of a product or service are directly valued by the respondent and composed ex-post into the overall utility (direct utility measurement using e.g., self-explicated approaches and analytic hierarchy process [AHP]; see Table 1). During a de-compositional preference measurement, a (whole) product or service with different characters is valued by the respondent and the part-worth for individual characteristics is deductively investigated (indirect utility measurement through e.g., conjoint analysis [CA]). The main difference between traditional or not choice-based CA and the choice-based CA (also called DCE) is that respondents rank or rate each alternative product or service defined in terms of their characteristics in the not choice-based CA. In contrast to this, respondents choose between two or more products or services in DCE (see Table 1). Beside compositional and de-compositional methods, a hybrid of both can be applied to elicit stated preferences in healthcare (e.g., adaptive conjoint analysis) [42, 45, 46].

Different patient and person preferences can be measured like therapy options as elements of healthcare. Regarding health state valuation – as a healthcare outcome – standard gamble (SG), time trade-off (TTO), and rating scales are the most widely used techniques to elicit health state preferences (e.g., to develop preference-based HRQoL instruments like EQ-5D). DCE is increasingly used in this context [11, 19]. Health state valuation methods and analysis depend on the selected elicitation technique (see Table 1).

#### Measuring PRO, PRE, and PRS

PRO, PRE, and PRS can be measured by qualitative, quantitative, and mixed methods. However, there is no standardized method specific to each. The following section will briefly introduce the methods that can be used by researchers for PRO, PRE, and PRS. The selection of methods to assess the patient- and person-reported constructs within a study depends on the research question and study population.

Regarding qualitative methods, PRO, PRE, and PRS can be assessed through different forms of interviews such as one-to-one interviews – semi-structured, guided, or open forms like narrative interviews – and focus groups [47]. Other qualitative methods include using open, semi-standardized, or computerized diaries as self-observational protocols (e.g., for reporting the occurrence of special events) [48, 49].

Applying quantitative methods, the most frequently used method to gain patient and person perspectives is a standardized self-reported questionnaire [50, 51]. Table 2 shows standardized self-administered instruments that can be used to assess PRO, PRE, and PRS. Qualitative methods are often also used as a first step to develop quantitative measures including standardized questionnaires. However, particularly in the field of PRS, healthcare organizations use individual, self-developed questionnaires rather than standardized questionnaires.

**Table 2:** Examples of standardized self-administered questionnaires for measuring PRO, PRE, and PRS

Category	Construct	Standardized questionnaire
Patient- and Person-Reported Outcomes (PRO)	Symptoms	Medical Outcome Study (MOS) Sleep Scale
		MD Anderson Symptom Inventory (MDASI)
	Functioning	WHO Disability Assessment Schedule 2.0 (WHODAS-2.0)
		Functional Status Questionnaires (FSQ)
	Well-being	Oxford Happiness Questionnaire (OHQ)
		Affected Balance Scale (ABS)
		Scale of Positive and Negative Experience (SPANE)
	HRQoL	EQ-5D
		Short-Form 36 (SF-36)
		World Health Organization Quality of Life Assessment (WHOQOL-100)
Patient- and Person-Reported Experiences (PRE)	Patient experiences	Patient Experience Questionnaire (PEQ)
		Improving Practices Questionnaire (IPQ)
		Patient Assessment Survey (PAS)
Patient- and Person-Reported Satisfaction (PRS)	Patient satisfaction	Patient Satisfaction Questionnaire (PSQ)
		European Project on Patient Evaluation of General Practice Care (EUROPEP) Questionnaire

Moreover, quantitative standardized interviews, standardized diaries, or diaries with statistics can be conducted [49, 51, 52]. A quantitative interview is based on a standardized questionnaire where the respondents are assisted by an interviewer who may give additional explanations. Some questionnaires like the EQ-5D can also be used as interviewer-administered standardized questionnaires, conducted either face-to-face or via telephone [51].

The method of analyzing patient- and person-reports depends on the assessment method; a qualitative method necessitates qualitative strategies for analyzing interviews or diaries (e.g., Mayring’s content analyses [53]), while for quantitative methods, statistical analysis like t-tests, non-parametric tests, or regressions using a statistics program have to be performed.

## Discussion

Although patient- and person-reports gain importance for evaluating healthcare systems and decision-making, a comprehensible categorization with a clear definition of constructs is still lacking. To bridge this gap the RHO Group presents a comprehensible and well-justified classification of patient- and person-reports of preferences, outcomes, experiences, and satisfaction in the healthcare setting.

In contrast to existing classification systems (e.g., Valderas & Alonso 2008 [5]), it embraces aspects of patient- and person-reports, incorporating several constructs, including also preferences that are gaining importance in healthcare. The categorization indicates the richness and diversity of patient- and person-reports.

While this classification is the first to consider all constructs reported by both patients as well as persons, including proxy-reports by caregivers, relatives, and the general population, it has some limitations. Any existing link between the four categories was deliberately excluded from the classification system by the RHO Group. Although the constructs are interrelated and interdependent, the objective of the new classification of preferences, outcomes, experiences, and satisfaction was to distinguish between the different concepts rather than illustrate all possible associations. A description that accounts for interrelations is beyond the scope of this research but should be considered in the future. Researchers should be aware of the links between the constructs, such as between satisfaction and outcome. Even though healthcare outcomes do not strongly affect the patient’s assessment of healthcare quality [40], low health status or chronic conditions negatively influence satisfaction [54]. Furthermore, satisfaction itself can influence outcomes. Higher patient satisfaction improves adherence [37]. Increased compliance to medical and non-medical treatments improves treatment outcomes [55]. Hence, patient satisfaction is linked to improved treatment outcomes through improved compliance.

Furthermore, differentiation of the four constructs and their dimensions is difficult; this differentiation depends on the constructs’ fundamental definitions and is therefore not selective. Moreover, the RHO Group decided to exclude the construct *adherence to treatment* from the present classification system as it depends not only on the individual, but also on the close cooperation between patient and practitioner and the influence of both. It was also excluded because it could not be separated from preferences, outcomes, satisfaction, and experiences. Moreover, it could not be incorporated within these categories because of the strong interrelations between adherence and these constructs. Hence, adherence could have influenced multiple constructs; for example, adherence could have influenced patient satisfaction and could have been influenced by patient’s satisfaction. As our aim was to provide a clear classification, rather than depict these interrelations, adherence was excluded. Nonetheless, patients’ adherence is an important concept and has a relevant impact on treatment outcomes [55, 56].

In addition to reorganizing constructs relating to existing patient- and person-reports, this paper also outlined the most constitutive methods for measuring such reports. These measures were first isolated and then grouped or structured. However, the grouping or structuring of the measures was difficult and was therefore not selective. The present classification system can be used when planning studies in health- and healthcare-related research. However, the choice of methods depends on the research aims. The (way of) results and interpretation will depend on the method used. Thus, methods for measuring patient- and person-reports should be carefully selected.

## Conclusions

This article aims at presenting a new and all-embracing classification system with well-defined constructs for reports related to health and healthcare provided by patients or persons. Measuring individuals’ preferences, outcomes, experiences, and satisfaction is gaining increasingly importance in health economics. Even though patient- and person-reports are always subjective and its ability to evaluate healthcare is often questioned, these reports comprise an essential and complementary part of patient- and person-centered healthcare. An overview of methods that can be used to measure the four categories of patient- and person-reports has been presented. While the value of understanding and using these reports in healthcare is increasingly recognized, its measurement and implementation presents a daunting challenge to researchers, clinicians, and patients [20]. Although there is a movement towards involving the client in healthcare policy-making, systematic and explicit consideration of research evidence on patient- and person-reported preferences, outcomes, experiences, and satisfaction in healthcare policy decisions seems to be still limited [1]. Future measurement of self-report in routine healthcare should be considered a standard process as it demonstrates long-term benefit for patients and the general population.

## Compliance with ethical standards and competing interests

This submission is a theoretical original research article and does not include data which were collected from human participants or animals. Therefore, no informed consent or ethics approval was required for this paper. All authors declare that they have no conflict of interest. They received no funding to develop the classification system and to prepare the manuscript.

## Abbreviations

ABS: affected balance scale
AHP: analytic hierarchy process
CA: conjoint analysis
CV: contingent valuation
DCE: discrete choice experiments
EUROPEP: European Project on Patient Evaluation of General Practice Care
FDA: Food and Drug Administration
FSQ: functional status questionnaires
HRQoL: health-related quality of life
ICF: international classification of functioning, disability and health
IPQ: improving practices questionnaire
LINMAP: linear programming technique for multidimensional analysis of preference
MDASI: MD Anderson Symptom Inventory
MONANOVA: monotonic analysis of variance
MOS: medical outcome study
OHQ: Oxford Happiness Questionnaire
OLS: ordinary least squares
PAS: patient assessment survey
PEQ: patient experience questionnaire
PRE: patient- and person-reported experiences
PREFMAP: preference mapping
PREMs: patient-reported experience measures
PRO: patient- and person-reported outcomes
PROM: patient-reported outcome measure
PRP: patient- and person-reported preferences
PRS: patient- and person-reported satisfaction
PSQ: patient satisfaction questionnaire
QoL: quality of life
RHO Group: Reported Health Outcomes Group
SF-36: Short-Form 36
SG: standard gamble
SPANE: scale of positive and negative experience
TTO: time trade-off
WHO: World Health Organization
WHODAS-2.0: World Health Organization Disability Assessment Schedule 2.0
WHOQOL-100: World Health Organization Quality of Life Assessment
WTA: willingness-to-accept
WTP: willingness-to-pay
